# Supplementary material for: Autoclaved Diet with Inactivated Spores of Bacillus spp. Decreased Reproductive Performance of Muc2−/− and Muc2+/− Mice
Source: Animals (Basel). 2022 Sep 13;12(18):2399. doi: 10.3390/ani12182399 (PMC9495189; doi:10.3390/ani12182399)
Supplement: Supplementary file 1 [file animals-12-02399-s001.zip › animals-1856579-supplementary.pdf]

Table S1. Primers used for the qPCR analysis.

Target	Primer Name	Primer Sequence 5' -> 3'
16S rRNA	*16S_F*<br>*16S_R*	TCCTACGGGAGGCAGCAG<br>ATTACCGCGGCTGCTGG
Akkermansia muciniphila	*AMUC_F*<br>*AMUC_R*	CAGCACGTGAAGGTGGGGAC<br>CCTTGCGGTTGGCTTCAGAT
Enterococcus faecalis	*Efec_F*<br>*Efec_R*	CGCTTCTTTCCTCCCGAGT<br>GCCATGCGGCATAAACTG
Lactobacillus murinus	*Lan_F*<br>*Lan_R*	GGCAATGATGCGTAGCCGAAC<br>CGCACTTTCTTCTCTAACAACAGG
Bacteroides spp.	*Bac sscp_F1*<br>*Bac sscp_R1*	CTG AAC CAG CCA AGT AGC GT<br>CGC AAT CGG AGT TCT TCG TG
Staphylococcus spp.	*Sspp1_F*<br>*Sspp1_R*	ATGCAAGTCGAGCGAAC(G/A)GA<br>TGTCTCAGTTCCAGTGTGGC
E.coli	*E. coli guaB_F*<br>*E. coli guaB_R*	TGCTTTCCGCAGCAATGGAT<br>CTGCGGATCAGTCACCACAC

Table S2. Data normality and statistical significance tests.

Parameters	Kolmogorov–Smirnov test, *p*	Mann–Whitney *U*-test, Z	*p*-value	Number of samples total
Age of prolapse (autoclaved v. non-autoclaved diets) of *Muc2*$^{-/-}$ females	$p < 0.001$	3.71	**0.0002**	43
**Feces from mice Muc2-/- on autoclaved diet v. feces from mice feeding of 2 weeks non-autoclaved diet**				
16S rRNA gene from *E. coli*	$p < 0.10$	2.12	**0.03**	7
16S rRNA gene from *Staphylococcus* spp.	$p > 0\ .10$	0.71	0.48	7
16S rRNA gene from *Bacteroides* spp.	$p > 0.10$	0.71	0.48	7
16S rRNA gene from *Lactobacillus murinus*	$p > 0.10$	-1.41	0.16	7
16S rRNA gene from *Enterococcus faecalis*	$p > 0.10$	0.35	0.72	7
16S rRNA gene from *Akkermansia muciniphila*	$p > 0.10$	-0.71	0.48	7
**Feces from mice Muc2-/- on autoclaved diet v. feces from mice feeding non-autoclaved diet**				
16S rRNA gene from *E. coli*	$p > 0.10$	1.64	0.10	8
16S rRNA gene from *Staphylococcus* spp.	$p > 0.10$	1.04	0.30	8
16S rRNA gene from *Bacteroides* spp.	$p < 0.05$	2.24	**0.02**	8
16S rRNA gene from *Lactobacillus murinus*	$p < 0.05$	2.24	**0.02**	8
16S rRNA gene from *Enterococcus faecalis*	$p > 0.10$	1.04	0.30	8
16S rRNA gene from *Akkermansia muciniphila*	$p > 0.10$	0.45	0.65	8
**Amount of immune cells, %**				
CD3+ cells in the blood of C57BL/6, %	$p > .10$	1.28	0.20	11
CD4+ cells in the blood of C57BL/6, %	$p < .01$	-2.74	**0.01**	11
CD8+ cells in the blood of C57BL/6, %	$p < 0.01$	2.74	**0.01**	11
CD19+ cells in the blood of C57BL/6, %	$p > 0.10$	-2.19	**0.03**	11
CD3+ cells in the blood of *Muc 2*$^{-/-}$, %	$p > 0.10$	0.00	1.00	9
CD4+ cells in the blood of *Muc 2*$^{-/-}$, %	$p > 0.10$	-1.72	0.09	9
CD8+ cells in the blood of *Muc 2*$^{-/-}$, %	$p > 0.10$	2.21	**0.03**	9
CD19+ cells in the blood of *Muc 2*$^{-/-}$, %	$p > 0.10$	1.23	0.22	9
CD3+ cells in the spleen of C57BL/6, %	$p < 0.03$	-2.61	**0.01**	10
CD4+ cells in the spleen of C57BL/6, %	$p > 0.10$	0.94	0.35	10
CD8+ cells in the spleen of C57BL/6, %	$p > 0.10$	0.52	0.60	10
CD19+ cells in the spleen of C57BL/6, %	$p < 0.03$	-2,61	**0.01**	10
CD3+ cells in the blood of *Muc 2*$^{-/}$, %	$p < 0.10$	-1,57	0.12	10
CD4+ cells in the spleen of *Muc 2*$^{-/-}$, %	$p < 0.10$	1,98	**0.05**	10
CD8+ cells in the spleen of *Muc 2*$^{-/-}$, %	$p < 0.10$	-1,57	0.12	10
CD19+ cells in the spleen of *Muc 2*$^{-/-}$, %	$p < 0.10$	-1,98	**0.05**	10

Table S3. Amino acid composition of the thigh muscle of *Muc2*$^{+/-}$ mice, %.

Substance	Non-autoclaved diet, mean±SEM	Autoclaved diet, mean±SEM	Kolmogorov–Smirnov test, *p*	Mann–Whitney *U*-test, Z	*p*-value	Number of samples total
Arginine	1.19±0.07	1.26±0.04	$p > 0.10$	-0.73	0.46	10
Lysine	1.37±0.06	1.45±0.04	$p > 0.10$	-0,73	0.46	10
Tyrosine	0.43±0.05	0.5±0.04	$p > 0.10$	-1,15	0.25	10
Phenylalanine	0.06±0.04	0.65±0.015	$p > 0.10$	-0,94	0.35	10
Histidine	0.35±0.03	0.35±0.01	$p > 0.10$	0.31	0.75	10
Leucine-isoleucine	1.89±0.32	2.27±0.04	$p > 0.10$	-0.73	0.46	10
Methionine	0.3±0.01	0.35±0.01	$p > 0.10$	-2,4	**0.02**	10
Valine	0.73±0.03	0.75±0.017	$p > 0.10$	0,0	1.00	10
Proline	0.53±0.01	0.54±0.01	$p > 0.10$	-0,31	0.75	10
Threonine	0.8±0.04	0.89±0.02	$p > 0.10$	-1,78	0.08	10
Serine	0.73±0.03	0.79±0.02	$p > 0.10$	-1,36	0.17	10
Alanine	0.88±0.04	0.9±0.02	$p > 0.10$	-0.94	0.35	10
Glycine	0.66±0.04	0.69±0.02	$p > 0.10$	0.73	0.46	10
Tryptophan	0.16±0.1	0.13±0.02	$p > 0.10$	1,04	0.3	10

Table S4. Analysis of the mineral and chemical composition of the diet, %.

Substance	Non-autoclaved diet, mean±SEM	Autoclaved diet, mean±SEM	Kolmogorov–Smirnov test, *p*	Mann–Whitney *U*-test, Z	*p*-value	Number of samples total
Arginine	0.79±0.04	0.92±0.05	$p > 0.10$	-1.53	0.13	6
Lysine	1.27±0.08	1.09±0.07	$p > 0.10$	1.53	0.13	6
Tyrosine	0.45±0.03	0.42±0.02	$p > 0.10$	0.65	0.51	6
Phenylalanine	0.52±0.03	0.44±0.03	$p > 0.10$	1.53	0.12	6
Histidine	0.34±0.02	0.4±0.03	$p > 0.10$	-1.53	0.13	6
Leucine-isoleucine	2.88±0.17	2.6±0.17	$p > 0.10$	0.65	0.51	6
Methionine	0.73±0.02	0.46±0.02	$p > 0.10$	1.96	**0.05**	6
Valine	1.22±0.07	1.1±0.07	$p > 0.10$	1.52	0.12	6
Proline	1.8±0.1	2.01±0.5	$p > 0.10$	0.65	0.51	6
Threonine	0.9±0.05	0.75±0.04	$p > 0.10$	1.53	0.13	6
Serine	1.22±0.08	1.01±0.06	$p > 0.10$	1.53	0.13	6
Alanine	1.2±0.07	1.05±0.6	$p > 0.10$	1.53	0.13	6
Glycine	1.13±0.07	1.08±0.06	$p > 0.10$	0.65	0.52	6
Glutamine	2.34±0.13	2.31±0.14	$p > 0.10$	0.65	0.52	6
Asparagine	1.09±0.07	1.27±0.08	$p > 0.10$	-1.53	0.12	6
Cystine	0.33±0.19	0.3±0.02	$p > 0.10$	0.65	0.52	6
Tryptophan	0.06±0.003	0.09±0.005	$p > 0.10$	1.96	**0.05**	6
Dry matter	87.8±5.3	86.84±5.01	$p > 0.10$	0.65	0.51	6
Moisture	9.08±3.92	13.24±0.69	$p > 0.10$	-0.65	0.51	6
Crude protein	21.36±1.35	19.38±1.06	$p > 0.10$	0.65	0.51	6
Crude fat	2.97±0.18	2.53±0.15	$p > 0.10$	1.53	0.12	6
Raw ash	6.17±0.19	4.05±0.1	$p > 0.10$	1.96	**0.05**	6
Crude fiber	2.76±0.16	2.46±0.14	$p > 0.10$	1.53	0.12	6
Sugar	0.85±0.06	0.71±0.04	$p > 0.10$	1.52	0.12	6
Starch	31±1.72	32.12±2.03	$p > 0.10$	-0.65	0.51	6
Calcium	0.83±0.05	0.75±0.05	$p > 0.10$	0.65	0.51	6
Phosphorus	0.61±0.04	0.51±0.03	$p > 0.10$	1.53	0.12	6

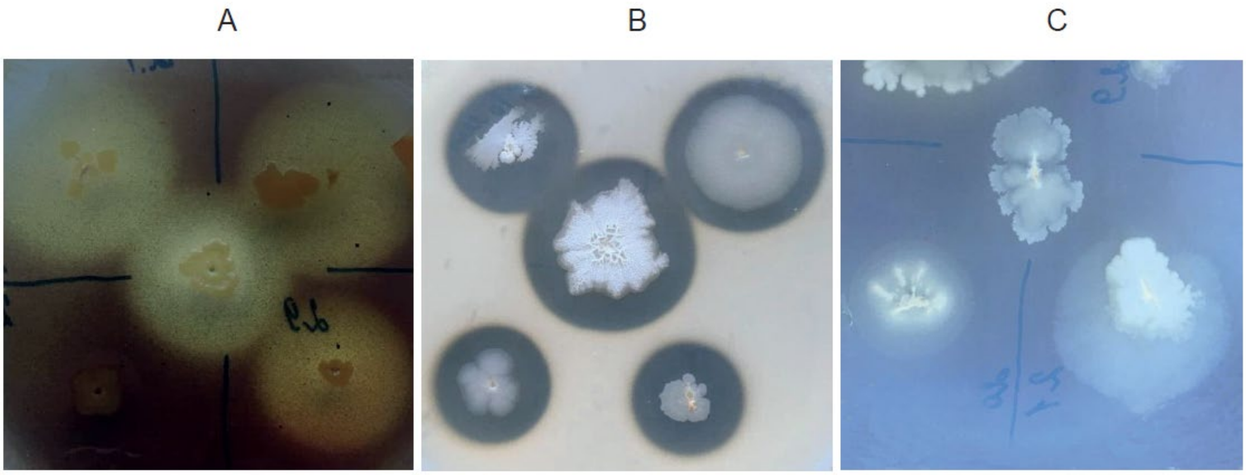

Figure S1. Enzymatic activity of bacterial strains from diet and feces. Qualitative test. (A) Amylolytic activity, (B) Proteolytic properties. (C) Lipase activity.
